# Direct Thrombectomy vs. Combined Treatment With Intravenous Thrombolysis in the Extended Time Window: A Target Trial Emulation

**DOI:** 10.1111/ene.70682

**Published:** 2026-07-01

**Authors:** Ettore Nicolini, Antonio Ciacciarelli, Giovanni Pracucci, Valentina Saia, Luigi Simonetti, Andrea Zini, Valerio Da Ros, Federica D'agostino, Marta Iacobucci, Manuela De Michele, Marco Andrighetti, Rossana Tassi, Andra Saletti, Ilaria Casetta, Enrico Fainardi, Alfredo Pauciulo, Marcella Caggiula, Roberto Menozzi, Alessandro Pezzini, Stefano Vallone, Guido Bigliardi, Domenico Sergio Zimatore, Marco Petruzzellis, Alessio Comai, Enrica Franchini, Luca Allegretti, Tiziana Tassinari, Nicola Limbucci, Patrizia Nencini, Giuseppe Carità, Monia Russo, Marco Filizzolo, Marina Mannino, Maria Ruggiero, Marco Longoni, Andrea Boghi, Andrea Naldi, Mauro Bergui, Giovanni Bosco, Giuseppe Pelle, Michele Alessiani, Matteo Alberti, Paolo Invernizzi, Raffaele Augelli, Manuel Cappellari, Guido Andrea Lazzarotti, Nicola Giannini, Daniel Konda, Fabrizio Sallustio, Salvatore Mangiafico, Danilo Toni, the IRETAS group

**Affiliations:** ^1^ Department of Human Neurosciences Sapienza University of Rome Rome Italy; ^2^ Stroke Unit, Policlinico Umberto I Sapienza University of Rome Rome Italy; ^3^ Department of NEUROFARBA, Neuroscience Section University of Florence Florence Italy; ^4^ Neurology and Stroke Unit S. Corona Hospital Pietra Ligure Italy; ^5^ IRCCS Istituto Di Scienze Neurologiche Di Bologna, Neuroradiology Unit Maggiore Hospital Bologna Italy; ^6^ IRCCS Istituto Di Scienze Neurologiche Di Bologna, Department of Neurology and Stroke Center Maggiore Hospital Bologna Italy; ^7^ Dipartimento Di Biomedicina E Prevenzione Università Degli Studi Di Roma Tor Vergata Rome Italy; ^8^ UOSD Stroke Unit Policlinico Tor Vergata Roma Italy; ^9^ Neuroradiology Unit, Umberto I Hospital, Department of Human Neurosciences Sapienza University Rome Italy; ^10^ UOC Stroke Unit AOU Senese Siena Italy; ^11^ UO Neuroradiologia Dip Neuroscienze AZOU Ferrara Ferrara Italy; ^12^ Neurology Unit University Hospital Arcispedale S. Anna Ferrara Italy; ^13^ Dipartimento Di Scienze Biomediche, Sperimentali E Cliniche, Neuroradiologia, Università Degli Studi Di Firenze Ospedale Universitario Careggi Firenze Italy; ^14^ Neuroradiologia Ospedale Vito Fazzi ASL LE Lecce Italy; ^15^ U.O.C Neurologia/U.O.S. Stroke Unit Ospedale Vito Fazzi Asl Lecce Lecce Italy; ^16^ Dipartimento Ad attività Integrata Interaziendale Diagnostico Azienda Ospedaliero Universitaria Parma Italy; ^17^ Dipartimento Di Medicina E Chirurgia, Università Degli Studi Di Parma ‐ Programma Stroke Care, Dipartimento Di Emergenza‐Urgenza Azienda Ospedaliero‐Universitaria Parma Italy; ^18^ Neuroradiologia, Ospedale Civile Di Baggiovara Azienda Ospedaliero‐Universitaria Di Modena Modena Italy; ^19^ Neurologia‐Stroke Unit, Ospedale Civile Di Baggiovara Azienda Ospedaliero‐Universitaria Di Modena Modena Italy; ^20^ Neuroradiologia AOU Consorziale Policlinico Bari Italy; ^21^ UOC Neurologia Universitaria E Stroke Unit “F. Puca” AOU Consorziale Policlinico Bari Italy; ^22^ Neuroradiologia, Ospedale Provinciale Di Bolzano (SABES‐ASDAA) Bolzano‐Bozen Italy; ^23^ Neurologia‐Stroke Unit Ospedale Provinciale Di Bolzano (SABES‐ASDAA) Bolzano‐Bozen Italy; ^24^ SC Neuroradiologia Osp. Santa Corona Pietra Ligure Asl 2 Sistema Sanitario Regione Liguria Pietra Ligure Italy; ^25^ SOD Interventistica Neurovascolare, AOU Careggi Firenze Italy; ^26^ SOD Stroke Unit Azienda Ospedaliero‐Universitaria Careggi Firenze Italy; ^27^ UOC Neuroradiologia Osp. Santa Maria Misericordia Rovigo Italy; ^28^ Stroke Unit ULSS5 Rovigo Italy; ^29^ UO Radiologia, A.O.O.R. Villa Sofia‐Cervello Palermo Italy; ^30^ UO Neurologia, A.O.O.R. Villa Sofia‐Cervello Palermo Italy; ^31^ Neuroradiologia Interventistica AUSL Romagna Cesena Cesena Italy; ^32^ UOC Neurologia E Stroke Unit ‐ Ospedale Bufalini Cesena‐ Azienda USL Della Romagna Cesena Italy; ^33^ Neuroradiologia Ospedale S.G. Bosco, ASL Città Di Torino Turin Italy; ^34^ Neurology Unit San Giovanni Bosco Hospital Turin Italy; ^35^ Dipartimento Di Neuroscienze Universitá Di Torino Torino Italy; ^36^ Stroke Unit AOU Citta Della Salute ‐ Ospedale Molinette Turin Italy; ^37^ Neuroradiologia Interventistica Latina Italy; ^38^ UOC Neurologia, Ospedale SM Goretti Latina Italy; ^39^ Neuroradiologia Fondazione Poliambulanza Di Brescia Brescia Italy; ^40^ Neurologia E Stroke Unit Fondazione Poliambulanza Di Brescia Brescia Italy; ^41^ Neuroradiologia ‐ Azienda Ospedaliera Universitaria Integrata Verona Verona Italy; ^42^ Stroke Unit ‐ Azienda Ospedaliera Universitaria Integrata Verona Verona Italy; ^43^ Neuroradiologia Azienda Ospedaliero Universitaria Pisana (AOUP) Pisa Italy; ^44^ UOC Neurologia ‐ Azienda Ospedaliero‐Universitaria Pisana (AOUP) Pisa Italy; ^45^ UOC Radiologia Diagnostica E Interventistica Ospedale Dei Castelli Asl Roma 6 Ariccia Italy; ^46^ Unità Di Trattamento Neurovascolare Ospedale Dei Castelli‐ASLRM6 Rome Italy; ^47^ IRCCS Neuromed Pozzilli (IS) Italy

**Keywords:** extended time window, intravenous thrombolysis, mechanical thrombectomy, stroke

## Abstract

**Background:**

In the early time window, direct mechanical thrombectomy (MT) is not non‐inferior to combined treatment with intravenous thrombolysis (IVT) for patients with large vessel occlusion (LVO) stroke, while its non‐inferiority in the extended time window remains uncertain. This study assessed whether direct MT is non‐inferior to IVT + MT beyond 4.5 h or at wake‐up.

**Methods:**

We emulated a non‐inferiority trial, comparing direct MT vs. IVT + MT, including patients with anterior circulation LVO between 4.5 and 24 h from symptom onset or at wake‐up, without contraindications to IVT and with target perfusion mismatch. We used inverse probability weighting (IPW) adjusted for pre‐specified covariates. The primary outcome was 90‐day mRS 0–2, with non‐inferiority defined by a lower 95% CI boundary of the Risk Difference (RD) ≥ −1.3%.

**Results:**

Among 347 patients, 212 received direct MT and 135 received IVT + MT. After IPW, patients treated with direct MT and IVT + MT had a similar likelihood of achieving a 90‐day mRS of 0–2 (adjRD –2.90 [95% CI –6.64 to 0.84]) with the lower boundary of the RD 95% CI crossing the non‐inferiority margin. Additionally, direct MT was associated with a shift toward a higher score on the 90‐day mRS (adjusted Common OR 1.59 [95% CI 1.05–2.39]), not confirmed after IPW, and with lower odds of successful recanalization (adjOR 0.38 [95% CI 0.18–0.78]). Rates of 90‐day mRS 0–1, sICH, and mortality were similar between groups.

**Conclusions:**

In our target trial emulation, direct MT was not non‐inferior to IVT + MT treatment beyond 4.5 h from symptom onset or at wake‐up, with IVT before MT yielding higher successful recanalization rates.

## Graphical abstract

This target‐trial emulation compared direct MT (*n* = 212) vs. IVT + MT (*n* = 135) in anterior‐circulation LVO patients treated 4.5–24 h or at wake‐up, eligible for IVT with perfusion mismatch. Using IPW with a non‐inferiority margin RD ≥ −1.3% for 90‐day mRS 0–2, direct MT was not non‐inferior to IVT + MT (adjRD −2.9%, 95% CI −6.64 to 0.84). IVT + MT yielded higher rates of successful recanalization; 90‐day mRS 0–1, sICH, and mortality were similar.

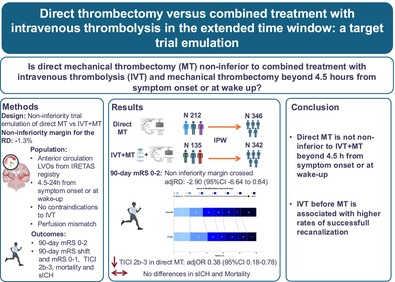

## 1 Introduction

Mechanical thrombectomy (MT) improves outcomes of patients with anterior cerebral circulation large vessel occlusion (LVO) treated beyond 6 h (h) from symptom onset or at wake‐up if selected with advanced neuroimaging [1]. Similarly, intravenous thrombolysis (IVT) with alteplase is effective in patients with acute ischemic stroke (AIS) with symptom onset at wake‐up or between 4.5 and 9 h [2, 3], and possibly up to 24 h from symptom onset, when perfusion neuroimaging reveals areas of salvageable penumbra [3, 4]. Indeed, despite the higher risk of symptomatic intracranial hemorrhage (sICH), IVT seems to be associated with a net clinical benefit between 4.5 and 24 h in terms of higher rates of favorable functional outcome [5].

Large randomized controlled trials did not show the non‐inferiority of direct MT compared to combined treatment with IVT within 4.5 h from symptom onset [6, 7, 8, 9], and a subsequent meta‐analysis showed better recanalization rates among patients treated with combined treatment [10].

It remains uncertain whether direct MT is as effective as combined IVT + MT treatment in patients with anterior cerebral circulation LVO in the extended time window. The TIMELESS trial did not demonstrate the benefit of IVT with Tenecteplase in this setting, although combined IVT + MT treatment appeared to be safe [11]. However, most of the patients were enrolled in comprehensive stroke centers (CSC) with short intervals between Tenecteplase administration and MT [11]. In contrast, observational data from patients admitted to primary stroke centers (PSC) and subsequently transferred to a CSC for MT suggest that combined treatment may benefit recanalization during transfer and functional outcomes at 90 days [12].

Considering the lack of randomized trials addressing this issue, in this study, we compared the outcomes of AIS patients treated with direct MT vs. combined IVT + MT treatment beyond 4.5 h from symptom onset or at wake‐up, by performing a target trial emulation from large national multicentric observational data.

## 2 Methods

### 2.1 Study Design, Setting, and Data Collection

We emulated a hypothetical non‐inferiority trial comparing outcomes of AIS patients with anterior circulation LVO treated with direct MT vs. IVT + MT beyond 4.5 h from symptom onset or at wake‐up. We used data from the Italian Registry of Endovascular Treatment in Acute Stroke (IRETAS) from January 2018 to December 2023; IRETAS is a multicenter observational registry of LVO patients treated endovascularly [13]. Additional registry details are reported elsewhere [13].

We categorized patients based on treatment: Direct MT or IVT (alteplase 0.9 mg/Kg) prior to MT. Neurologists and neuroradiologists collected clinical and radiological data.

Ischemic core was defined at magnetic resonance imaging (MRI) (apparent diffusion coefficient [ADC] ≤ 620 × 10^−6^ mm^2^/s) or at perfusion CT (pCT) (relative cerebral blood flow [rCBF] < 30% of that in normal tissue). Critically hypoperfused tissue was defined as a region with a maximum (Tmax) delay of more than 6 s either on perfusion weighted imaging MR or on pCT. The difference between Tmax > 6 s and rCBF < 30% or ADC ≤ 620 × 10^−6^ mm^2^/s (mismatch) was considered ischemic penumbra, and their ratio was defined as the mismatch ratio. Neuroradiologists assessed recanalization at the end of the procedure, according to the Thrombolysis in Cerebral Infarction (TICI) score [14], and hemorrhagic transformation at 24 h on CT or MR images.

Our analysis was reported according to the TrAnsparent ReportinG of Studies Emulating a Target trial (TARGET) Guideline.

### 2.2 Participants

We selected patients treated with MT between 4.5 and 24 h from known symptom onset, or from last seen well for wake‐up or unknown onset, in accordance with current guidelines [15, 16]; specifically, those meeting the clinical and radiological criteria of the DAWN [17] and DEFUSE‐3 [18] trials. Patients were treated with IVT before MT at wake‐up or between 4.5 and 9 h from known symptom onset in accordance with current guidelines recommending IVT for cases that meet the clinical and radiological criteria of the EXTEND and WAKE‐UP trials [19, 20]. Finally, patients were treated with IVT before MT between 9 and 24 h, outside the current guidelines [15, 16], based on the judgment of the vascular neurologists at each participating center. All patients included in the final analysis met the following criteria: (1) older than 16 years of age; (2) AIS from anterior cerebral circulation LVO (M1 or M2 segment of middle cerebral artery [MCA], or intracranial segment of internal carotid artery [ICA] or Tandem occlusion); (3) Perfusion neuroimaging at baseline showing an ischemic core volume < 70 mL, a hypoperfusion volume > 10 mL and a mismatch ratio > 1.2;^2^ (4) Onset‐to‐Imaging time beyond 4.5 h and within 24 h or symptom onset at wake‐up. We excluded patients with: (1) any contraindication to IVT according to current guidelines,^16^ except for time window (see Table S1 for a list of absolute and relative contraindications to IVT); (2) concomitant posterior circulation LVO (3) pre‐stroke modified Rankin scale (mRS) score > 2; (4) ischemic core volume ≥ 70 mL or hypoperfusion volume ≤ 10 mL or mismatch ratio ≤ 1.2 (4) missing data on functional outcome at 90 days.

### 2.3 Outcomes

The primary efficacy outcome was 90‐day mRS 0–2 (good functional outcome). The secondary efficacy outcomes included: (1) ordinal shift at the 90‐day mRS; (2) 90‐day excellent functional outcome, defined as mRS 0–1; (3) successful recanalization, defined as a TICI score 2b–3. Safety outcomes included: (1) 90‐day mortality; (2) sICH according to European Cooperative Acute Stroke Study (ECASS) II criteria [21].

### 2.4 Statistical Analysis

Continuous variables were presented as median (interquartile range, IQR) or mean (standard deviation, SD), as appropriate; categorical variables were presented as numbers and percentages. Student's *t*‐test or Mann–Whitney U test was used for continuous data. Pearson's χ2 test or Fisher's exact test was used for categorical data as appropriate.

For the primary outcome, the non‐inferiority margin was set at a risk difference (RD) of −1.3% [10]. Therefore, we prespecified that non‐inferiority would be met if the lower 95% CI boundary of the RD was superior to or equal to −1.3%. The non‐inferiority margin of −1.3% for the lower boundary of the 95% CI of the RD was selected based on previous methods from the European Stroke Organization guidelines group, as well as from the Improving Reperfusion Strategies in Ischemic Stroke (IRIS) Collaborators [10, 22]. This margin was deemed an acceptable minimal clinically important difference by a large cohort of experts [23].

The primary analysis compared rates of 90‐day good functional outcome (mRS 0–2) between direct MT and IVT + MT groups.

We applied inverse probability weighting (IPW) to adjust for confounding. A logistic regression model was fitted with direct mechanical thrombectomy (direct MT) as the outcome, conditional on prespecified covariates; covariates included all variables with *p* < 0.10 for between‐group differences in univariate analyses and variables reported in the literature to influence treatment choice (age, sex, wake‐up onset, hypertension, diabetes, hypercholesterolemia, history of TIA or stroke in the last 3 months, atrial fibrillation, smoking habit, baseline NIHSS, direct access to a thrombectomy‐capable center, core volume, and site of occlusion). The causal structure of our analysis was represented with the use of a directed acyclic graph (DAG) (Figure S1). We used the model's estimated probabilities to compute stabilized IPW [24], which were then used as weights to derive the weighted population. The weighted sample size, therefore, depends on each subject's stabilized weight, such that subjects with larger weights contribute more to the weighted sample.

For the primary outcome analysis, we used a robust variance estimator adjusted for prespecified covariates: Age, sex, baseline NIHSS, wake‐up onset, imaging‐to‐recanalization time, atrial fibrillation, diabetes, arterial hypertension, site of occlusion, and infarct‐core volume. These covariates were selected from variables with *p* < 0.10 for association with the outcome in univariate analyses and from established predictors in the literature. The robust variance estimator was applied to account for possible duplication of observations introduced by the IPW procedure.

We also estimated differences in rates for all secondary outcomes and the odds of a worse functional outcome using an ordinal analysis of the 90‐day mRS between treatment groups. RDs were calculated as the difference between the mean predicted probabilities of the outcome in each treatment group; 95% confidence intervals were derived from the variances of the predicted probabilities.

We additionally estimated the difference in the rates of the primary outcome across the following pre‐specified subgroups: Age, NIHSS, time strata, site of occlusion, and access modality (direct to a CSC vs. transfer from a PSC).

We performed two sensitivity analyses: First, excluding patients treated beyond 9 h from known symptom onset; second, testing non‐inferiority with odds ratios (OR) margins of 0.8 and 0.74, as used in DIRECT‐MT [25] and SKIP [8] trials, respectively. Furthermore, we repeated outcome analysis, including also patients who did not fulfill the prespecified perfusion criteria.

No Imputation was performed based on the assumption that data were not missing at random. Probability values < 0.05 were considered statistically significant. All analyses were conducted using SPSS (V.30) software (IBM).

### 2.5 Ethics Statement

The study conformed to the Declaration of Helsinki. Ethical approval or patient consent varied among centers. Informed consent for data use was obtained from all patients.

## 3 Results

### 3.1 Patient Characteristics

**FIGURE 1:**
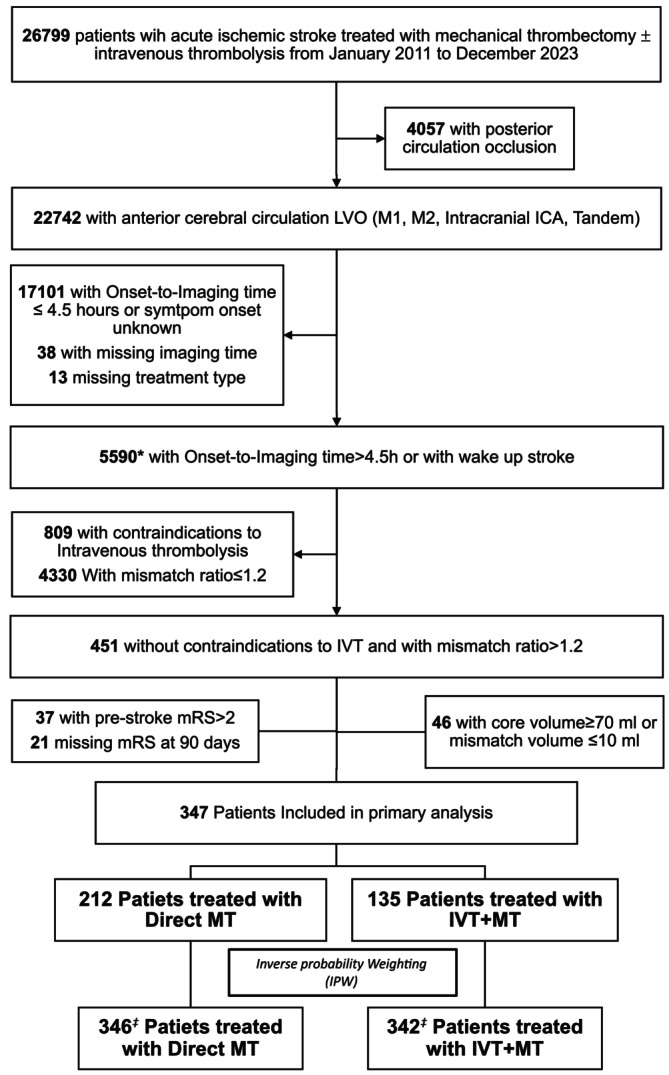
Study Cohort and Emulated Trial Flow. *Legend*: ICA, internal carotid artery; IVT, intravenous thrombolysis; LVO, large vessel occlusion; mRS, modified Rankin Scale; MT, mechanical thrombectomy. *All treated from 2018 onward; ‡ Weighted sample size.

Out of 5590 patients with anterior circulation LVO and onset‐to‐imaging time between 4.5 and 24 h or with wake‐up onset, 347 with complete 90‐day follow‐up data were included for the analysis (Figure 1, Table S4). The mean (±SD) age was 74.4 (±12.7) years; 202 (54.9%) patients were female. A total of 212 (61.1%) patients were treated with direct MT, while 135 (38.9%) received IVT before MT (Table 1).

**TABLE 1:** Baseline patient characteristics before and after applying IPW.

Characteristic	Original cohort		*p*	Weighted cohort		*p*	SMD
	Treatment group			Treatment group			
	Direct MT (*N* 212)	Combined IVT + MT (*N* 135)		Direct MT (*N* 346)^a^	Combined IVT + MT (*N* 342)^a^		
Age Mean ± SD	73.9 ± 12.8	74.6 ± 12.5	0.633	74.3 ± 12.8	74.4 ± 12.6	0.699	0.029
Male	90 (42.5)	65 (48.1)	0.298	158 (45.6)	159 (46.6)	0.828	0.020
Female	122 (57.5)	70 (51.9)		188 (54.4)	183 (53.4)		
History of TIA/Stroke^b^	9 (4.2)	1 (0.7)	0.057	10 (2.9)	4 (1.2)	0.110	0.118
Atrial fibrillation	60 (28.3)	22 (16.3)	0.010	83 (24.0)	78 (22.8)	0.714	0.027
Diabetes	31 (14.6)	25 (18.5)	0.336	53 (15.4)	51 (14.8)	0.882	0.016
Hypertension	151 (71.2)	97 (71.9)	0.900	245 (70.7)	243 (71.1)	0.944	0.010
Current tobacco use	34 (16.0)	11 (8.1)	0.033	47 (13.4)	55 (16.0)	0.357	0.071
Hypercholesterolemia	62 (29.2)	58 (43.0)	0.009	114 (32.9)	117 (34.1)	0.746	0.025
Wake‐up stroke	98 (46.2)	68 (50.4)	0.451	167 (48.2)	169 (49.4)	0.763	0.025
Direct access to CSC	160 (75.5)	119 (88.1)	0.004	279 (80.5)	285 (83.2)	0.318	0.071
Transfer from PSC	52 (24.5)	16 (11.9)		68 (19.5)	57 (16.8)		
NIHSS Median (IQR)	15 (8–20)	14 (9–19)	0.768	15 (8–20)	14 (9–19)	0.503	0.051
MRI	12 (5.7)	3 (2.2)	0.125	20 (5.7)	6 (1.7)	0.006	0.215
CTp	200 (94.3)	132 (97.8)		326 (94.3)	336 (98.3)		
ASPECTS Median (IQR)^d^	8 (7–9)	9 (8–10)	< 0.001	8 (7–10)	9 (8–10)	< 0.001	0.364
Core volume, ml Median (IQR)	8 (0–24)	5 (0–19)	0.057	7 (0–22)	5 (0–20)	0.706	0.029
Hypoperfusion Volume ml Median (IQR)	96 (61–130)	84 (50–131)	0.541	92 (61–127)	90 (56–131)	0.507	0.051
Onset‐to‐CT min Median (IQR)^c^	526 (360–771)	452 (323–639)	0.309	553 (363–771)	447 (323–627)	0.037	0.223
Onset‐to‐Needle (*N* = 69)^c^	—	500 (370–668)	—	—	499 (351–668)	—	
Onset‐to‐groin, min Median (IQR)^c^	635 (470–875)	560 (435–800)	0.117	640 (470–875)	555 (450–748)	0.004	0.312
Door‐to‐groin, min Median (IQR)	103 (80–131)	110 (87–138)	0.997	105 (81–128)	113 (87–139)	0.541	0.047
Needle‐to‐groin, min Median (IQR)	—	55 (41–76)		—	55 (42–76)	—	
CT‐to‐Recanalization, min Median (IQR)	140 (97–190)	133 (110–181)	0.442	140 (95–186)	141 (110–190)	0.936	0.032
Duration of the procedure, min Median (IQR)	50 (29–93)	45 (30–70)	0.095	49 (29–92)	49 (30–70)	0.368	0.069
Site of Occlusion (DSA)							
Intracranial ICA	44 (20.8)	17 (12.6)	0.049	63 (18.2)	61 (17.7)	0.973	0.025
M1	80 (37.7)	49 (36.3)		129 (37.2)	126 (36.9)		
M2	64 (30.2)	52 (38.5)		113 (32.6)	110 (32.0)		
Tandem Occlusion	21 (9.9)	10 (7.4)		31 (8.9)	36 (10.5)		
No occlusion	3 (1.4)	7 (5.2)		11 (3.1)	10 (2.9)		
Extracranial balloon angioplasty or stenting^e^	27 (20.3)	10 (11.4)	0.082	40 (18.8)	37 (6.5)	0.493	0.059
Periprocedural complication	34 (16.0)	22 (16.3)	0.949	47 (13.7)	61 (17.8)	0.125	0.113
Vessel perforation	9 (4.2)	3 (2.2)	0.315	14 (4.0)	15 (4.5)	0.818	0.027
Arterial dissection	3 (1.4)	0	0.165	3 (0.9)	0	0.084	0.138
Thrombus migration	15 (7.1)	7 (5.2)	0.481	20 (5.8)	20 (6.0)	0.970	0.008
Puncture site hematoma	1 (0.5)	6 (4.4)	0.010	2 (0.5)	14 (4.0)	0.002	0.238
Other	7 (3.3)	6 (4.4)	0.585	10 (2.9)	11 (3.3)	0.804	0.021

*Note:* Data are presented as mean ± SD, number (%), or median (IQR).

Abbreviations: ASPECTS, Alberta Stroke Program Early CT Score; CSC, comprehensive stroke center; ICA, internal carotid artery; IPW, inverse probability weighting; IQR, interquartile range; IVT, intravenous thrombolysis; MT, mechanical thrombectomy; NIHSS, National Institutes of Health Stroke Scale; PSC, primary stroke center; SD, standard deviation; SMD, standardized mean differences; TIA, transient ischemic attack.

^a^ Weighted sample size.

^b^ History of TIA/Stroke within the last 3 months.

^c^ Just patients with known symptom onset.

^d^ Data available for 286 patients.

^e^ Data available for 221 patients.

After applying IPW, we obtained a cohort of 346 patients in the direct MT group and 342 patients in the IVT + MT group. Further details on the distribution of baseline characteristics in both treatment groups before and after IPW are reported in Table 1.

### 3.2 Efficacy Outcomes

**FIGURE 2:**
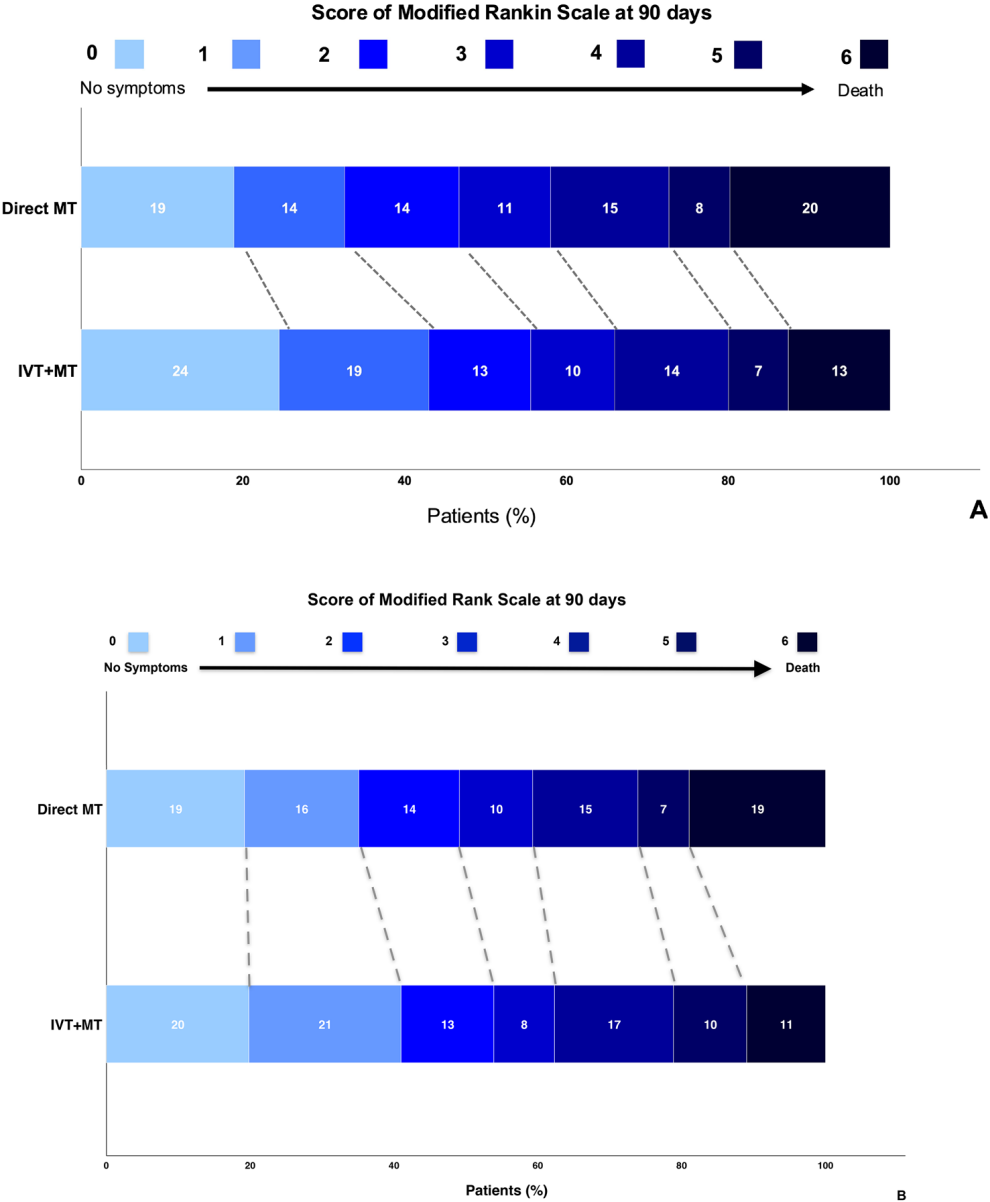
Modified Rankin Scale (mRS) Scores at 90 Days according to treatment groups before (A) and after (B) IPW. *Legend*: Grotta bar‐chart showing the distribution and percentage of each mRS score at 90 days among patients treated with direct MT vs. combined IVT + MT, (A) before and (B) after IPW. Abbreviations: mRS, modified Rankin Scale; MT, Mechanical Thrombectomy; IVT, Intravenous thrombolysis; IPW, inverse probability weighting.

In the original cohort, 99 (46.7%) patients from the direct MT group and 75 (55.6%) in the IVT + MT group achieved a 90‐day mRS score 0–2 (Figure 2A) (adjusted odds ratio [aOR] 0.67 [95% CI 0.41–1.19]). After IPW, 170 (49.0%) direct MT‐treated and 178 (51.9%) IVT + MT‐treated patients achieved a 90‐day mRS score 0–2 (Figure 2B), the adjusted risk difference (AdjRD) was −2.90 (95% CI −6.64 to 0.84) with the lower boundary of the 95% CI crossing the prespecified non‐inferiority margin of −1.3%. After IPW, 119 (34.3%) direct MT‐treated patients and 138 (40.2%) IVT + MT‐treated patients achieved a 90‐day mRS score 0–1 (aOR 0.70 [95% CI 0.40–1.20]) (Table 2).

**TABLE 2:** Primary and secondary outcomes analysis direct MT‐treated vs. IVT + MT–treated patients before and after IPW.

	Original cohort				Weighted cohort	
	Treatment group		*p*	Adjusted odds ratio (95% CI)^a^	Adjusted odds ratio (95% CI)^a^	Adjusted risk difference, % (95% CI)
	Direct MT (*N* = 212)	Combined IVT + MT (*N* = 135)				
Primary outcome						
Modified Rankin scale score of 0–2 at 90 days	99 (46.7)	75 (55.6)	0.108	0.67 (0.41–1.19)	0.89 (0.53–1.52)	−2.90 (−6.64 to 0.84)
Secondary outcomes						
mRS score at 90 days						
0	40 (18.9)	33 (24.4)	0.515	1.59 (1.05–2.39)^b^	1.27 (0.84–1.91)^b^	—
1	29 (13.7)	25 (18.5)				
2	30 (14.2)	17 (12.6)				
3	24 (11.3)	14 (10.4)				
4	31 (14.6)	19 (14.1)				
5	16 (7.5)	10 (7.4)				
6	42 (19.8)	17 (12.6)				
mRS score 0–1 at 90 days	69 (32.5)	58 (43.0)	0.050	0.58 (0.34–0.98)	0.70 (0.40–1.20)	−5.90 (−9.57 to −2.23)
Successful recanalization (TICI 2b‐3)	183 (80.6)	125 (91.2)	0.006	0.39 (0.19–0.82)	0.38 (0.18–0.78)	−9.50 (−11.77 to −7.23)
Safety outcome						
Death at 90 days	42 (19.8)	17 (12.6)	0.081	1.84 (0.90–3.76)	1.94 (0.92–4.09)	7.20 (5.28 to 9.12)
Symptomatic ICH (ECASS II criteria)	13 (5.8)	8 (6.0)	0.932	0.84 (0.32–2.20)	1.01 (0.36–2.84)	0.30 (−0.56 to 1.16)

Abbreviations: ICH, intracerebral hemorrhage; IPW, inverse probability weighting; IVT, intravenous thrombolysis; MT, mechanical thrombectomy; TICI, thrombolysis in cerebral infarction.

^a^ The value was adjusted for Age, Sex, baseline NIHSS, onset at wake‐up, imaging‐to‐recanalization time, atrial fibrillation, diabetes, arterial hypertension, site of occlusion, and volume of the infarct core.

^b^ Adjusted common odds ratio.

Before IPW, direct MT was associated with a shift toward a higher score at the 90‐day mRS (adjusted common OR 1.59 [95% CI 1.05–2.39]), while no difference was observed after IPW. Additionally, the direct MT group showed lower odds of achieving successful recanalization (TICI 2b‐3) (aOR 0.38 [95% CI 0.18–0.78]) (Table 2).

In the prespecified sensitivity analysis excluding patients treated beyond 9 h from known symptom onset, out of 267 patients, 72 (45.9%) direct MT‐treated and 64 (58.2%) IVT + MT‐treated reached 90‐day mRS 0–2 (AdjRD after IPW −4.80 [95% CI −9.15 to −0.45]). Moreover, direct MT was associated with lower odds of 90‐day mRS 0–1 in the unweighted cohort (aOR 0.46 [95% CI 0.26–0.84]), but this association was not confirmed after IPW (aOR 0.56 [95% CI 0.31–1.01]) (Table S2).

**FIGURE 3:**
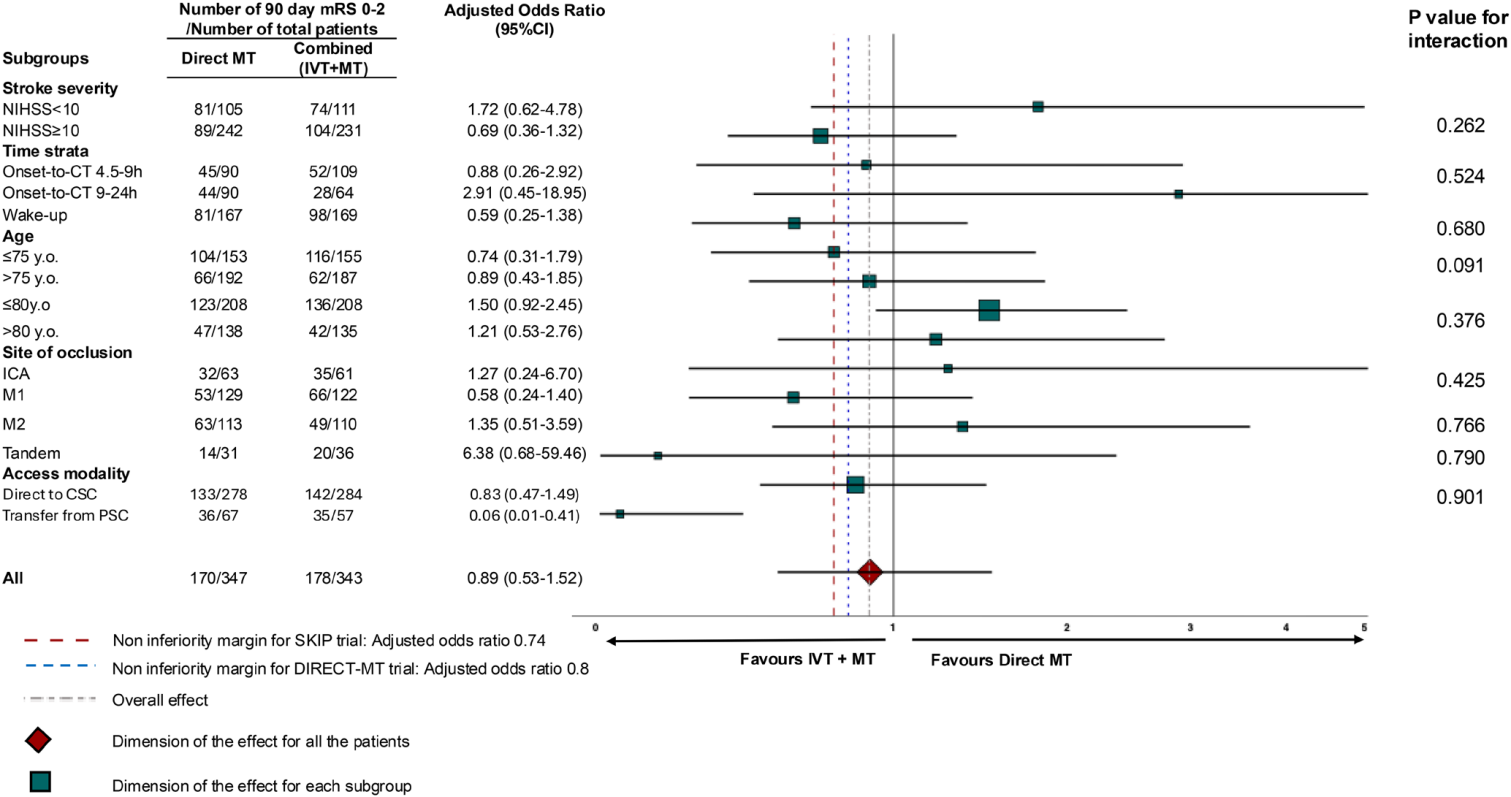
Subgroup Analyses for 90‐day favorable outcome with different non‐inferiority margins. Legend: Forest Plot showing the adjusted odds ratios for 90‐day favorable outcome (mRS 0–2) in pre‐specified subgroups. There were no significant differences between direct MT and IVT + MT in the tested subgroups. Adjusted odds ratio for all patients 0.89 (95% CI 0.53–1.52) with lower boundary of the 95% CI crossing the non‐inferiority margins of 0.8 and 0.74, as defined for DIRECT‐MT and SKIP trials. Abbreviations: NIHSS, National Institutes of Health Stroke Scale; mRS, modified Rankin Scale; h, hours; MT, Mechanical Thrombectomy; IVT, Intravenous thrombolysis; y.o., years old.

The association between MT and 90‐day mRS 0–2 was similar across subgroups defined by stroke severity, time strata, age, site of occlusion, and access modality, without significant interactions (Figure 3).

### 3.3 Safety Outcomes

Mortality was similar between groups within the original cohort (aOR 1.84 [95% CI 0.90–3.76]), as well as after applying IPW (aOR 1.94 [95% CI 0.92–4.09]).

After IPW, in the direct‐MT group, 20 (5.8%) patients had sICH according to ECASS II, as compared to 19 (5.7%) patients who received IVT + MT (aOR 1.01 [95% CI 0.36–2.84]) (Table 2).

## 4 Discussion

In this targeted trial emulation on patients with anterior circulation LVO beyond 4.5 h from symptom onset or at wake‐up, direct MT was not non‐inferior to combined treatment with IVT + MT in terms of 90‐day functional independence (mRS 0–2). Moreover, IVT before MT was associated with higher rates of successful recanalization (TICI 2b–3). We did not find any safety difference between direct‐MT and IVT + MT, either for 90‐day mortality or for sICH occurrence, despite a trend for higher mortality in the direct‐MT group.

We chose to design our study for non‐inferiority instead of superiority, considering the high efficacy of both MT and IVT separately in the extended time window [1, 2]. Additionally, Italian guidelines recommend as a good practice point the use of both IVT and MT in this time window when patients are eligible for both of them [15]. Therefore, the non‐inferiority design, as previously done in the early time window [6, 7, 8, 25, 26], seemed the most appropriate for assessing the real‐world use of direct‐MT and IVT + MT in this setting. Our results support the treatment of selected stroke patients with both IVT and MT in the extended time window. Four large trials failed to show the non‐inferiority of direct MT within 4.5 h from symptom onset [6, 8, 9, 27], while two trials with larger non‐inferiority margins concluded for the non‐inferiority of direct MT [25, 26]. A subsequent meta‐analysis of the six trials, [6, 7, 8, 9, 25, 26] confirmed that direct MT is not non‐inferior to combined IVT + MT treatment. The authors set the non‐inferiority margin of −1.3% for the lower boundary of the 95% CI of the RD [10], which was considered as an acceptable minimal clinically important difference among a large cohort of experts [23]. Accordingly, in our targeted trial emulation, we adopted the same non‐inferiority margin. Actually, there is no agreement for the best non‐inferiority margin in stroke trials; the −1.3% margin might be considered too strict, while larger non‐inferiority margins might be too generous [28]. Considering different non‐inferiority margins, our study would show the non‐inferiority of direct‐MT according to the DEVT [26] trial (margin of 10% of the RD), while non‐inferiority would not be met with the margins set at 0.8 (DIRECT‐MT [25]) and 0.74 (SKIP [8]) of the aOR (Figure 3) as well as with the margin of 5% of the RD as was set for the individual participant data meta‐analysis from the IRIS collaboration group [29].

Similarly to the observations in the early time window [10, 27], combined IVT + MT treatment led to higher rates of successful recanalization in our patients. The better recanalization status of the IVT + MT group might have contributed to the functional outcome in our cohort, though other authors highlighted the benefit of IVT before MT irrespective of recanalization status [30].

Another meta‐analysis highlighted an apparent loss of benefit of IVT before MT beyond 140 min from symptom onset [22]. Differently from the studies in the early time window, we analyzed a population with a favorable perfusion pattern. Indeed, a tissue‐based selection of patients might overcome the effect of time on functional outcome. Previous reports from large IVT registries have shown how tissue‐based criteria perform better than the conventional time window in the selection of patients who benefit from IVT [31].

Our data support reperfusion treatment with IVT + MT beyond the early 4.5 h time window, and potentially beyond 9 h, whenever perfusion neuroimaging highlights salvageable tissue. This opportunity of effective and safe combined therapy in the extended time windows seems even more possible with the use of Tenecteplase, according to recent trials [32, 33, 34]. Overall, our results align with large trials showing that direct MT is not non‐inferior to combined treatment in the early time window [6, 7, 8, 9, 25, 26]. However, different from the early time window, accurate patient selection through perfusion neuroimaging is essential when considering candidates for both IVT and MT beyond 4.5 h from known symptom onset or at wake‐up.

Notably, a large proportion of patients were excluded from our analysis because of incomplete perfusion data or because they did not fulfill the prespecified perfusion criteria. We performed a supplementary analysis including these patients, which showed the non‐inferiority of direct MT as compared to combined IVT + MT treatment (Table S3). We believe that the advantage of direct MT in this enlarged cohort might be related to the lack of defined perfusion criteria, reducing the efficacy of IVT associated with MT. Indeed, large trials showed clearly the benefit of alteplase in the extended time window for patients meeting perfusion mismatch criteria, while the same benefit was not evident in patients without perfusion mismatch [2].

In our study, patients were treated within a median time of 7.7 h with IVT and 9.5 h with MT. As a matter of fact, a small proportion of our patients were treated with IVT + MT beyond 9 h from symptom onset, and our results might not be generalizable as regards this underrepresented subgroup. Further studies with a larger proportion of patients treated between 9 and 24 h are needed.

We also performed a prespecified analysis excluding patients treated beyond 9 h from symptom onset, which showed a trend against direct MT for the primary outcome, since the upper limit of the 95% CI for the adjusted RD was lower than zero [35]. Moreover, in this subgroup, patients treated with IVT+ MT had higher rates of 90‐day excellent functional outcome. These results may suggest a greater benefit of IVT prior to MT when treatment occurs earlier.

We found similar effects of direct MT across all the subgroups tested. Of note, the subgroup of patients admitted to a PSC and transferred to a CSC showed a trend toward greater benefit from combined treatment, in line with the study from Seners et al. that highlighted better outcomes at 90 days, as well as higher rates of recanalization during transfer among patients admitted to PSCs who received IVT before MT [12]. On the contrary, the TIMELESS trial did not demonstrate the benefit of combined treatment with Tenecteplase compared to direct MT in patients with anterior circulation LVO, selected with perfusion imaging in the extended time window [11]. Most patients from the TIMELESS trial were directly admitted to thrombectomy‐capable centers with short intervals between Tenecteplase administration and MT. Similarly, in our study, more than 80% of patients were directly admitted to a thrombectomy‐capable center, which may explain why we could not find any superiority of the combined treatment. The proportion of patients first admitted to a PSC was higher in the Direct MT group, which may have introduced treatment time differences between groups; however, PSC patients remained a minority in the Direct MT cohort. We found shorter door‐to‐groin times and longer onset‐to‐needle times among PSC patients, while other time metrics (e.g., CT‐to‐recanalization) were similar across access‐modality groups (Table S6). Finally, from a tissue‐based perspective, perfusion‐imaging‐to‐recanalization times were similar between Direct MT and IVT + MT groups, suggesting that any treatment‐time differences by access modality likely had a minimal effect on the observed between‐group differences.

In our cohort, the two tested treatments were associated with similar rates of sICH, similar to the findings of the main trials on this argument in the early time window. About 6% of our patients experienced a clinically significant hemorrhagic transformation, a percentage similar to that reported in the extended time window when testing MT [1] and IVT [2] separately. Considering also the similar rates of mortality, our study outlines the safety of IVT + MT beyond 4.5 h.

This study has some limitations. First, direct MT and IVT + MT groups differed at baseline before applying IPW. The baseline disparities among treatment groups were well‐balanced after applying IPW but should be considered as potential sources of bias when interpreting our results. In particular, onset‐to‐treatment times were longer among patients treated with direct MT, which may have favored infarct progression and worse clinical outcomes in this group, although this applies to only half of our cohort (patients with known symptom onset). However, all our patients were selected based on perfusion imaging, which revealed similar core volumes among the treatment groups. Therefore, imaging‐to‐recanalization times, which were also similar between the groups, might be more relevant for assessing the risk of infarct progression in our cohort. Furthermore, we adjusted our multivariate analysis for imaging‐to‐recanalization time to reduce the risk of bias due to time delay metrics, but time differences should be considered when interpreting our results. Furthermore, patients treated with IVT + MT had a higher ASPECTS as compared to the direct MT group, even after applying IPW, which might have influenced the outcome of our patients [36]. Nevertheless, core volume at baseline did not differ significantly among treatment groups, suggesting similar extension of the ischemic damage. Despite adjustment for multiple confounders, residual confounding from the observational design and unmeasured variables may introduce immortal bias affecting our analysis and should be considered when interpreting the results.

Second, we chose to include wake‐up stroke patients, regardless of the hypothetical time of symptom onset, which might provoke higher heterogeneity in our population. Large trials considered last time seen well [17] or midtime of sleep [20] as a hypothetical time of symptom onset on an arbitrary basis, while we chose to select wake‐up patients through a tissue‐based perfusion one.

Third, perfusion imaging was acquired and analyzed through different protocols and software in each center (Table S5), with a risk of over‐ or underestimation of core and hypoperfusion volumes. Indeed, our patients had small core volumes, and our results might not be generalizable to other patients with larger core volumes.

Fourth, none of our patients were treated with Tenecteplase, which might lead to better outcomes compared to alteplase [34].

Finally, the decision to treat patients with or without IVT before MT may vary across centers, potentially introducing selection bias that should be considered when interpreting our results.

Our study has several strengths. First, the design of the target trial emulation allows adjusting the observational data for confounders by indication, thereby preventing selection bias. This approach allows us to gain stronger evidence compared to traditional observational studies and is a useful method when randomized trials are lacking [37, 38]. Second, our study is based on a large cohort of patients selected with defined mismatch criteria. Third, our data were adjusted for several confounders and evaluated across different subgroups.

## 5 Conclusions

In patients with AIS from anterior circulation LVO with evidence of salvageable tissue in the extended time window, direct MT was not non‐inferior to IVT + MT, and IVT + MT treatment was associated with higher rates of successful recanalization. The study suggests that patients in the extended time window, selected through perfusion neuroimaging, can be effectively and safely treated with both treatments.

## Author Contributions

**Alessandro Pezzini:** investigation. **Antonio Ciacciarelli:** conceptualization, writing – review and editing, methodology, investigation. **Valentina Saia:** project administration, writing – review and editing, data curation. **Manuela De Michele:** investigation. **Giovanni Pracucci:** writing – review and editing, project administration, data curation. **Marta Iacobucci:** investigation. **Guido Andrea Lazzarotti:** investigation. **Nicola Giannini:** investigation. **Luigi Simonetti:** investigation. **Ettore Nicolini:** conceptualization, investigation, methodology, writing – original draft, formal analysis. **Valerio Da Ros:** investigation. **Andrea Zini:** investigation, writing – review and editing. **Alfredo Pauciulo:** investigation. **Rossana Tassi:** investigation. **Marcella Caggiula:** investigation. **Ilaria Casetta:** investigation. **Roberto Menozzi:** investigation. **Andra Saletti:** investigation. **Luca Allegretti:** investigation. **Guido Bigliardi:** investigation. **Domenico Sergio Zimatore:** investigation. **Marco Andrighetti:** investigation. **Patrizia Nencini:** investigation. **Stefano Vallone:** investigation. **Enrica Franchini:** investigation. **Giuseppe Carità:** investigation. **Tiziana Tassinari:** investigation. **Enrico Fainardi:** investigation. **Nicola Limbucci:** investigation. **Monia Russo:** investigation. **Maria Ruggiero:** investigation. **Federica D'agostino:** investigation. **Daniel Konda:** investigation. **Marco Filizzolo:** investigation. **Marco Longoni:** investigation. **Marina Mannino:** investigation. **Raffaele Augelli:** investigation. **Giovanni Bosco:** investigation. **Fabrizio Sallustio:** investigation, writing – review and editing. **Andrea Naldi:** investigation. **Alessio Comai:** investigation. **Marco Petruzzellis:** investigation. **Giuseppe Pelle:** investigation. **Michele Alessiani:** investigation. **Danilo Toni:** project administration, writing – review and editing, conceptualization, methodology, supervision. **Matteo Alberti:** investigation. **Mauro Bergui:** investigation. **Paolo Invernizzi:** investigation. **Salvatore Mangiafico:** investigation, writing – review and editing, project administration, supervision. **Andrea Boghi:** investigation. **Manuel Cappellari:** investigation, writing – review and editing.

## Funding

This work was supported by Ministero della Salute, RFPS‐2006‐1‐336562.

## Disclosure

The authors have nothing to report.

## Ethics Statement

The present study is in accordance with the ethical standards laid down in the 1964 Declaration of Helsinki and its later amendments. This study involves human participants and was approved by Comitato Etico Regionale per la Sperimentazione Clinica della Regione Toscana (ID: FS‐MIP).

## Consent

Informed consent to use anonymized and aggregated data for participation in the IRETAS was obtained from all patients of each center.

## Conflicts of Interest

The authors declare no conflicts of interest.

## Supporting information


**Table S1:** Absolute and relative contraindications to intravenous thrombolysis according to current guidelines.
**Table S2:** Outcomes analysis for patients treated at 4.5–9 h from known symptom onset or at wake‐up, before and after IPW.
**Table S3:** Outcomes analysis, including patients not fulfilling the perfusion criteria or with incomplete perfusion data, before and after IPW.
**Table S4:** Missing values within the entire population and in each treatment group.
**Table S5:** Perfusion software used for core and penumbra volumes evaluation of the entire population and in each treatment group.
**Table S6:** Time metrics according to access modality before and after IPW.
**Figure S1:** Directed acyclic graph.

## Data Availability

The data that support the findings of this study are available from the corresponding author upon reasonable request.
