# Supplementary material for: Direct Thrombectomy vs. Combined Treatment With Intravenous Thrombolysis in the Extended Time Window: A Target Trial Emulation
Source: Eur J Neurol. 2026 Jul 1;33(7):e70682. doi: 10.1111/ene.70682 (PMC13323805; doi:10.1111/ene.70682)

**Supplemental Data**

Content list

- **Table S1.** Absolute and relative contraindications to intravenous thrombolysis according to current guidelines
- **Table S2.** Outcomes analysis for patients treated at 4.5-9 hours from known symptom onset or at wake up, before and after IPW
- **Table S3.** Outcomes analysis including patients not fulfilling the perfusion criteria or with incomplete perfusion data, before and after IPW
- **Table S4.** Missing values within the entire population and in each treatment group.
- **Table S5.** Perfusion software used for core and penumbra volumes evaluation the entire population and in each treatment group.
- **Table S6.** Time metrics according to access modality before and after IPW
- **Figure S1.** Directed acyclic graph

**Table S1. Absolute and relative contraindications to intravenous thrombolysis according to current guidelines**

| **Absolute and relative contraindications to intravenous thrombolysis** |
| --- |
| **Absolute contraindications** |
| Intracranial hemorrhage on brain CT scan |
| Clinical suspicion of SAH, even if CT is normal |
| Administration of intravenous heparin within the previous 24 hours and aPTT exceeding the upper normal limit of the laboratory |
| Recent (≤ 24 hours) intake of direct anticoagulant drug not antagonizable in the absence of specific tests |
| Platelet count < 100,000/mm3 |
| Known hemorrhagic diathesis |
| Ongoing or recent severe bleeding |
| Suspicion of ongoing intracranial hemorrhage |
| Bacterial endocarditis, pericarditis |
| Acute pancreatitis |
| Neoplasm with increased hemorrhagic risk |
| Severe liver disease, including liver failure, cirrhosis, portal hypertension (esophageal varices), active hepatitis |
| Hemorrhagic retinopathy |
| High hemorrhagic risk due to comorbidities |
| Recent (< 10 days) traumatic external cardiac massage, delivery, puncture of non-compressible blood vessel (e.g., subclavian or jugular vein) |
| Ulcerative disease of the gastrointestinal tract (< 3 months) |
| Diagnosis of amyloid angiopathy according to Boston criteria 2.0^6^ |
| **Relative contraindications** |
| Minor neurological deficit or rapidly improving symptoms prior to the start of the infusion |
| Severe stroke clinically assessed (NIHSS > 25) and/or through appropriate neuroimaging techniques |
| Seizure at the onset of the stroke |
| Stroke in the 3 months preceding the event |
| Systolic blood pressure > 185 mmHg or diastolic > 110 mmHg, or aggressive treatment necessary to reduce blood pressure within these limits |
| Blood sugar <50 or >400 mg/dl |
| History of central nervous system pathology (e.g. neoplasm, aneurysm, intracranial or spinal surgery) |

**Table S2. Outcomes analysis for patients treated at 4.5-9 hours from known symptom onset or at wake up, before and after IPW**

|  | **Before IPW** | | | | **After IPW** | |
| --- | --- | --- | --- | --- | --- | --- |
|  | **Treatment group** | |  |  |  |  |
|  | **Direct MT** | **Combined**  **IVT+MT** | **P** | **Adjusted Odds Ratio (95% CI)†** | **Adjusted Odds Ratio (95%CI) †** | **Adjusted Risk difference,% (95%CI)** |
| **Primary outcome** | | | | | | |
| Modified Rankin scale score of 0–2 at 90 days | 72  (45.9) | 64  (58.2) | 0.047 | 0.51 (0.28-0.94) | 0.67 (0.37-1.22) | -4.80 (-9.15 to -0.45) |
| **Secondary outcomes** | | | | | | |
| Modified Rankin scale score of 0–1 at 90 days | 52  (33.1) | 49  (44.5) | 0.058 | 0.46 (0.26-0.84) | 0.56 (0.31-1.01) | -5.90 (-10.06 to -1.74) |
| Successful recanalization (TICI 2b-3) | 141  (83.4) | 101  (91.7) | 0.071 | 0.50 (0.23-1.10) | 0.46 (0.21-1.04) | -4.9 (-7.37 to -2.43) |
| **Safety outcomes** | | | | | | |
| Death at 90 days | 31  (19.7) | 12  (10.9) | 0.053 | 2.22 (0.89-5.51) | 2.24 (0.95-5.30) | 8.00 (5.89 to 10.11) |
| Symptomatic ICH (ECASS II criteria) | 8  (4.7) | 5  (4.5) | 0.942 | 0.99 (0.29-35) | 1.08 (0.35-3.30) | -0.10 (-1.09 to 0.89) |

† The value was adjusted for age, sex, baseline NIHSS, onset at wake-up, imaging-to-recanalization time, atrial fibrillation, diabetes, arterial hypertension, site of occlusion and volume of the infarct core.

**Table S3. Outcomes analysis including patients not fulfilling the perfusion criteria or with incomplete perfusion data, before and after IPW**

|  | **Before IPW** | | | | **After IPW** | | | |  |
| --- | --- | --- | --- | --- | --- | --- | --- | --- | --- |
|  | **Treatment group** | |  |  | **Treatment group** | |  |  |  |
|  | **Direct MT**  **(N 2401)** | **Combined**  **IVT+MT (N 836)** | **P** | **Adjusted Odds Ratio (95% CI)†** | **Direct MT**  **(N 3155)** | **Combined IVT+MT (N 3305)** | **P** | **Adjusted Odds Ratio (95%CI) †** | **Adjusted Risk difference,% (95%CI)** |
| **Primary Outcome** | | | | | | | | | |
| Modified Rankin scale score of 0–2 at 90 days | 1160 (48.3) | 444 (53.1) | 0.018 | 0.99 (0.81-1.22) | 1537 (48.7) | 1702 (51.5) | 0.027 | 1.03 (0.84-1.27) | 0.02 (0.00, 0.03) |
| **Secondary Outcomes** | | | | | | | | | |
| Modified Rankin scale score of 0–1 at 90 days | 803 (33.4) | 324 (38.8) | 0.006 | 1.03 (0.83-1.29) | 1069 (33.9) | 1235 (37.4) | 0.004 | 1.03 (0.83-1.28) | 0.01 (-0.01, 0.02) |
| Successful recanalization (TICI 2b-3) | 2055 (79.8) | 738 (86.7) | <0.001 | 0.61 (0.47-0.79) | 2696 (80.0) | 2912 (86.5) | <0.001 | 0.62 (0.48-0.81) | -0.04 (-0.05, -0.02) |
| **Safety Outcomes** | | | | | | | | | |
| Death at 90 days | 423 (17.6) | 104 (12.4) | <0.001 | 0.72 (0.55-0.95) | 539 (17.1) | 445 (13.5) | <0.001 | 0.78 (0.59-1.05) | -0.01 (-0.02, 0.01) |
| Symptomatic ICH (ECASS II criteria) | 180 (7.1) | 59 (6.9) | 0.877 | 0.88 (0.61-1.26) | 230 (6.9) | 252 (7.5) | 0.925 | 0.86 (0.59-1.24) | -0.01 (-0.03, 0.01) |

† The value was adjusted for age, sex, baseline NIHSS, onset at wake-up, imaging-to-recanalization time, atrial fibrillation, diabetes, arterial hypertension, site of occlusion and ASPECT score at admission.

**Table S4. Title:** Missing values within the entire population and in each treatment group.

|  | **Valid cases** | **Missing cases** | | |
| --- | --- | --- | --- | --- |
|  | **All**  **N (%)** | **All**  **N (%)** | **Direct MT**  **N (%)** | **IVT + MT**  **N (%)** |
| Age | 347 (100) | 0 | 0 | 0 |
| Sex | 347 (100) | 0 | 0 | 0 |
| History of TIA/Stroke | 347 (100) | 0 | 0 | 0 |
| Atrial fibrillation | 347 (100) | 0 | 0 | 0 |
| Diabetes | 347 (100) | 0 | 0 | 0 |
| Hypertension | 347 (100) | 0 | 0 | 0 |
| Current tobacco use | 347 (100) | 0 | 0 | 0 |
| Hypercholesterolemia | 347 (100) | 0 | 0 | 0 |
| Wake-up stroke | 347 (100) | 0 | 0 | 0 |
| NIHSS | 347 (100) | 0 | 0 | 0 |
| Core volume, ml Median (IQR) | 347 (100) | 0 | 0 | 0 |
| Hypoperfusion Volume ml Median (IQR) | 347 (100) | 0 | 0 | 0 |
| Onset-to-CT^(1)^, min Median (IQR) | 347 (100) | 0 | 0 | 0 |
| Onset-to-Needle^(1)^ (N=69) | 347 (100) | 0 | 0 | 0 |
| Onset-to-groin^(1)^, min Median (IQR) | 347 (100) | 0 | 0 | 0 |
| CT-to-Recanalization, min Median (IQR) | 347 (100) | 0 | 0 | 0 |
| Duration of the procedure, min Median (IQR) | 347 (100) | 0 | 0 | 0 |
| Periprocedural complication | 347 (100) | 0 | 0 | 0 |
| Modified Rankin scale at 90 days | 347 (94.3) | 0 | 0 | 0 |
| TICI score | 347 (100) | 4 (1.1) | 3 (1.3) | 1 (0.7) |
| sICH | 347 (100) | 8 (2.2) | 4 (1.7) | 4 (2.9) |

**Table S5. Title:** Perfusion software used for core and penumbra volumes evaluation the entire population and in each treatment group.

|  | **All**  **N (%)** | **Direct MT**  **N (%)** | **IVT + MT**  **N (%)** |
| --- | --- | --- | --- |
| Rapid AI | 225 (61.1) | 141 (61.3) | 84 (60.9) |
| Siemens syngo.via | 79 (21.5) | 53 (23.0) | 26 (18.8) |
| GE ADW | 24 (6.5) | 16 (7.0) | 8 (5.8) |
| Olea | 9 (2.4) | 6 (2.6) | 3 (2.2) |
| Brainomix | 31 (8.4) | 14 (6.1) | 17 (12.3) |

**Table S6. Title:** Time metrics according to access modality before and after IPW

|  | **Original cohort** | |  | **Weighted cohort** | |  |
| --- | --- | --- | --- | --- | --- | --- |
|  | **Tranfer from PSC**  **(N 68)** | **Direct access to CSC (279)** | **p** | **Tranfer from PSC (N**^‡^ **125)** | **Direct access to CSC (N**^‡^  **564)** | **p** |
| Onset-to-CT^†^ min Median (IQR) | 500 (331–771) | 497 (346–701) | 0.808 | 500 (363–679]) | 480 (341–687) | 0.490 |
| Onset-to-Needle^†^ (N=69) | 668 (370-814) | 475 (356-600) | 0.037 | 668 (370-814) | 460 (351-593) | 0.009 |
| Onset-to-groin^†^, min Median (IQR) | 613 (441–842) | 609 (469–835) | 0.702 | 613 (441–800) | 582 (467–810) | 0.432 |
| Door-to-groin, min Median (IQR) | 81 (59–97) | 110 (89–138) | <0.001 | 84 (63–106) | 110 (90–138) | <0.001 |
| Needle-to-groin, min Median (IQR) | 49 (42-54) | 57 (41-56) | 0.560 | 49 (42-54) | 57 (43-76) | 0.610 |
| CT-to-Recanalization, min Median (IQR) | 122 (94-175) | 144 (104-189) | 0.638 | 125 (95-178) | 146 (109-190) | 0.263 |

^‡^ Weighted sample size; † Just patients with known symptom onset

Abbreviations: IPW, Inverse probability weighting; CSC, comprehensive stroke center; PSC, primary stroke center; IQR, Interquartile range; min, minutes.

**Figure S1. Directed acyclic graph**


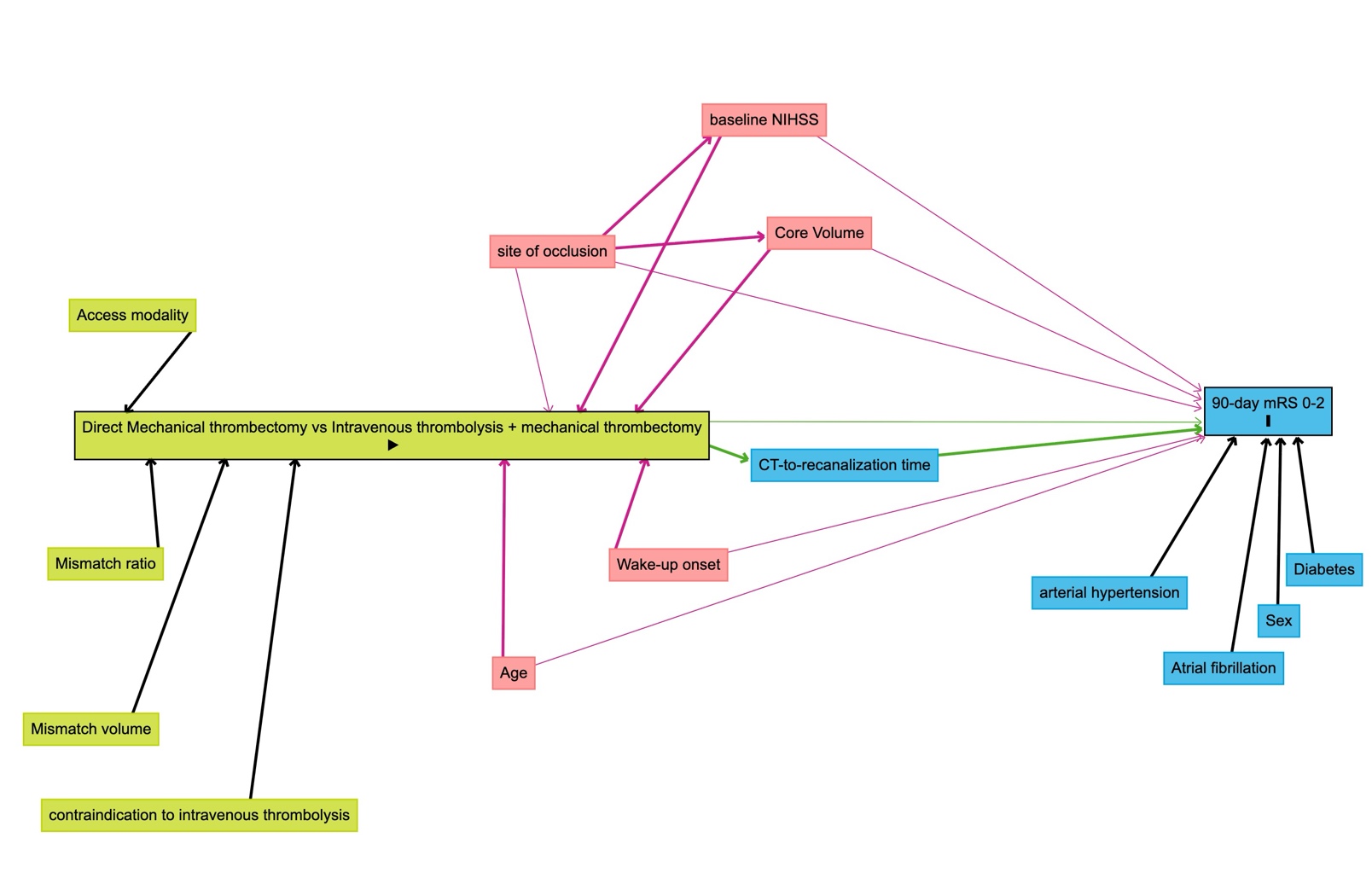

Supplement: Supplementary file 1 — Table S1: Absolute and relative contraindications to intravenous thrombolysis according to current guidelines. Table S2: Outcomes analysis for patients treated at 4.5–9 h from known symptom onset or at wake‐up, before and after IPW. Table S3: Outcomes analysis, including patients not fulfilling the perfusion criteria or with incomplete perfusion data, before and after IPW. Table S4: Missing values within the entire population and in each treatment group. Table S5: Perfusion software used for core and penumbra volumes evaluation of the entire population and in each treatment group. Table S6: Time metrics according to access modality before and after IPW. Figure S1: Directed acyclic graph. [file ENE-33-e70682-s001.docx]
